# Enhancing bone marrow regeneration by SALL4 protein

**DOI:** 10.1186/1756-8722-6-84

**Published:** 2013-11-05

**Authors:** Wenbin Liao, Jerell R Aguila, Yixin Yao, Jianchang Yang, Gary Zieve, Yongping Jiang, Cecilia Avila, Lisa Senzel, Raymond Lai, Dazhong Xu, Wei Dai, Yupo Ma

**Affiliations:** 1 Department of Pathology, University Hospital, Stony Brook University, Stony Brook, NY 11794, USA; 2 Department of Surgery, University Hospital, Stony Brook University, Stony Brook, NY 11794, USA; 3 Department of Obstetrics & Gynecology, University Hospital, Stony Brook University, Stony Brook, NY 11794, USA; 4 Department of Environmental Medicine, New York University Langone Medical Center, 57 Old Forge Road, Tuxedo, NY 10987, USA; 5 Department of Laboratory Medicine and Pathology, University of Alberta and Cross Cancer Institute, Edmonton, Alberta T6G1Z2, Canada; 6 Biopharmaceutical Research Center, Chinese Academy of Medical Sciences & Peking Union Medical College, Suzhou 215126, China

**Keywords:** Hematopoietic stem cell, Regeneration, Bone marrow transplantation, SALL4, Recombinant protein

## Abstract

Hematopoietic stem cells (HSCs) are widely used in transplantation therapy to treat a variety of blood diseases. The success of hematopoietic recovery is of high importance and closely related to the patient’s morbidity and mortality after Hematopoietic stem cell transplantation (HSCT). We have previously shown that SALL4 is a potent stimulator for the expansion of human hematopoietic stem/progenitor cells in vitro. In these studies, we demonstrated that systemic administration with TAT-SALL4B resulted in expediting auto-reconstitution and inducing a 30-fold expansion of endogenous HSCs/HPCs in mice exposed to a high dose of irradiation. Most importantly, TAT-SALL4B treatment markedly prevented death in mice receiving lethal irradiation. Our studies also showed that TAT-SALL4B treatment was able to enhance both the short-term and long-term engraftment of human cord blood (CB) cells in NOD/SCID mice and the mechanism was likely related to the in vivo expansion of donor cells in a recipient. This robust expansion was required for the association of SALL4B with DNA methyltransferase complex, an epigenetic regulator critical in maintaining HSC pools and in normal lineage progression. Our results may provide a useful strategy to enhance hematopoietic recovery and reconstitution in cord blood transplantation with a recombinant TAT-SALL4B fusion protein.

## Background

Hematopoietic stem cell transplantation (HSCT) is a type of stem cell therapy used to treat cancers such as lymphoma and leukemia and other blood-related diseases. However, there have been several limitations in the use of bone marrow hematopoietic cell transplantation. The primary limitation is that the donor pool is limited by the need for at least partial HLA matching and it usually takes time in finding a suitable donor [1]. Human umbilical cord blood (hUCB) is increasingly being used as an alternative source of hematopoietic stem/progenitor cells (HSC/HPC) for allogeneic HSCT because of its rapid availability and less stringent requirement for HLA matching [2]. This is especially important for minority patients and patients of mixed ethnicity, where hUCB is a particularly attractive alternative donor stem cell source. However, the absolute number of hUCB HSC/HPC transplanted is much lower than bone marrow or mobilized peripheral blood stem cells due to the limited volume of hUCB. This leads to significantly delayed engraftment and increased peri-transplant complications [3-6]. One approach to overcome this problem is to use two unrelated hUCB units for the transplantation [7,8]. While this strategy improved adult engraftment rates, it brought about worse GVHD (graft-versus-host disease) [9]. In addition, of two or more hUCBs transplanted into one recipient, it is usual for only one of those multiple hUCBs infused in the patient to be present [7,8,10]. There are multiple steps critical for HSC/HPC engraftment in vivo including homing to a niche, and then the seeded HSC/HPC expand and proliferate, or engraft. Several other strategies that are being explored to increase the engraftment include ex vivo expansion of hUCB HSC/HPC [11-14], increase of hUCB HSC/HPC homing by CD26 inhibitor or SDF-1/CXCR4 regulation, and addition of third party mesenchymal stem cells. However, there are still problems like risk of contamination and loss of long-term engraftable cells in the process of ex vivo manipulation of hUCB involved in these strategies [15]. The identification of agents to increase hUCB engraftment by in vivo expansion of transplanted hUCB HSC/HPC without in vitro manipulation is of significant therapeutic value.

Recently, we demonstrated that lentivirus expression of SALL4 in human bone marrow HSC/HPC was able to dramatically expand these cells and enhance their ability of long-term engraftment in NOD/SCID mice [16-18]. In order to evaluate in vivo effect of SALL4 on hematologic recovery and the engraftment of donor cell in HSCT, we produced SALL4B protein in baculovirus expression vector system (BEVS) and injected the protein into animals after iradiation or HSCT. In present study, we successfully expressed and isolated a TAT-SALL4B fusion protein carrying the protein transduction domain of HIV transactivating protein (TAT) in BEVS and showed the activity of the recombinant TAT-SALL4B protein in vitro. In addition, the TAT-SALL4B accelerates the regeneration of mouse bone marrow after lethal or sub-lethal irradiation by in situ expansion of the bone marrow HSC/HPC. In a transplantation model where human cord blood CD34+ cells were introduced into NOD/SCID mouse, TAT-SALL4B protein treatment is able to augment both short-term and long-term engraftment of human cells in the recipient mice. Our results suggest the potential utility of recombinant TAT-SALL4B protein as a stimulator for hematologic recovery after myelosuppression and an enhancer for the engraftment of cord blood cells in HSCT.

## Results

### Expression and purification of TAT-SALL4B fusion protein

**Figure 1:**
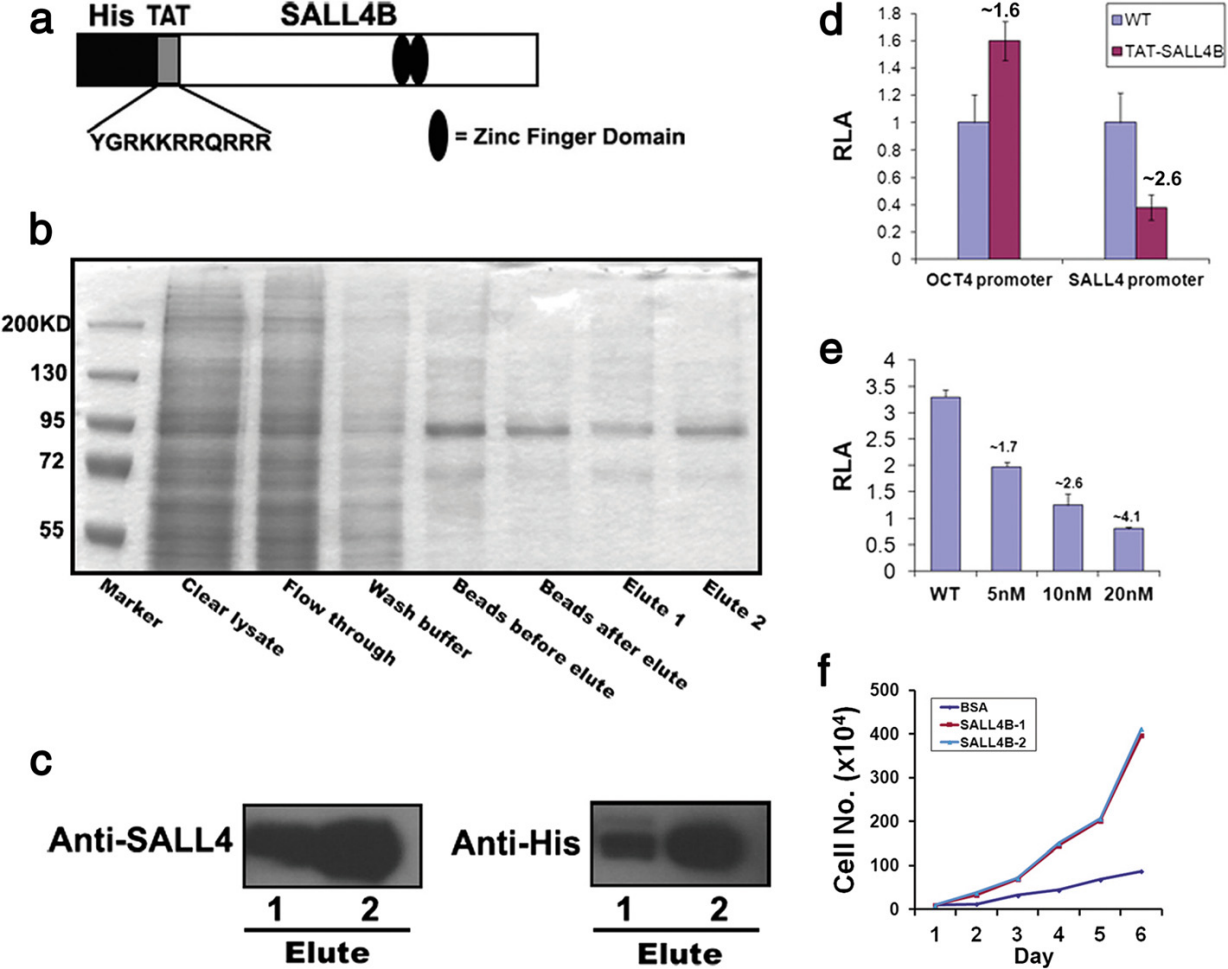
**Purification and in vitro activity of TAT-SALL4B protein. (a)**: Schematic diagram of SALL4B construct for TAT-SALL4B fusion protein expression in insect cells. **(b)**: SDS-PAGE gel picture of Purified TAT-SALL4B, the size of TAT-SALL4B is ~95 KD. **(c)**: Western blots showed TAT-SALL4B fusion protein could be recognized by SALL4 and or Histidine (His) antibodies. **(d)**: TAT-SALL4B protein regulates Oct4 and SALL4 promoter activities as showed in a luciferase report assay. Protein concentrations were 5 nM (OCT4 promoter) and 10 nM (SALL4-promoter) separately. WT RLA was standardized to 1. **(e)**: The inhibitory effect of TAT-SALL4B protein on SALL4 promoter activity is dose dependent. *Note:* Fold changes compared to WT were labeled on top of the diagrams. RLA: Relative Luciferase Activity; WT: Wide Type. **(f)**: TAT-SALL4B protein promotes the proliferation of mouse LSK cells in culture.

Previously, we demonstrated that SALL4-tranduced human CD34+ cells were capable of rapid expansion *in vitro*[16]. Protein transduction utilizing cell penetrating peptides (CPPs) can overcome many of the limitations of lentiviral vectors. Therefore, we sought to develop a CPP SALL4B fusion protein, TAT-SALL4B expressed in a Sf9 insect cell system. We then tested the impact of TAT-SALL4 on the growth of residual bone marrow cells in vivo after ablation. The SALL4 stem cell gene contains two alternatively spliced isoforms. SALL4A is the large spliced variant and SALL4B is a smaller variant with approximately half the full-length of SALL4A. We focused our studies on SALL4B [16] because it is a shorter form and expressed a high level in SF9 cells. In Figure 1a, the structures and functional module of the SALL4B construct for the baculovirus expression system are shown. The full-length TAT-SALL4B was expressed in baculovirus-infected Sf9 cells and purified using anti-Histidine (His) affinity chromatography (Figure 1b-c).

### Functional activity of TAT-SALL4B fusion protein in vitro

In our earlier experiments utilizing SALL4B lentiviral transduction [19], we showed that SALL4B could regulate the expression of multiple genes involved in the self-renewal maintenance of human ES cells including OCT4 and Nanog. In addition, SALL4B can regulate its own promoter [20]. In order to test if TAT-SALL4B bears similar functional activities, we constructed OCT4 and SALL4 luciferase promoter reporters into 293 T cells, we found TAT-SALL4B protein could regulate OCT4 and SALL4 promoter activities in a similar pattern to that reported in previous study [19] (Figure 1d). The TAT-SALL4B protein significantly upregulated OCT4 and downregulated SALL4 promoter activities. The luciferase activities also showed a dose dependent manner in the presence of TAT-SALL4B protein. (Figure 1e). We also tested the activity of TAT-SALL4B protein on the growth of mouse bone marrow HSCs/HPCs enriched by a combination of Lineage, c-Kit and Sca-1 magnetic cell sorting. LSK (Lin-/c-Kit+/Sca-1+) cells were treated with 20 nM TAT-SALL4B or BSA control. After 6 days of culture, the total cell number was increased by ~40 fold in the TAT-SALL4B group as compared to only ~8 fold in the control (Figure 1f).

### Enhancing bone marrow recovery by TAT-SALL4B

**Figure 2:**
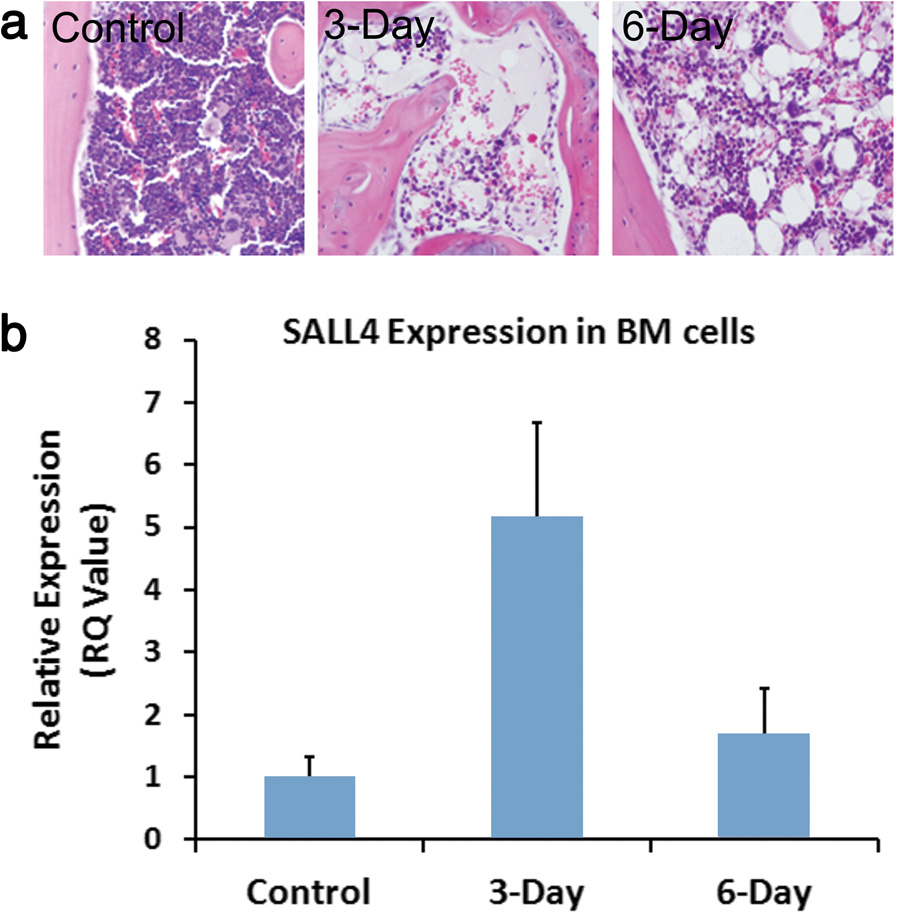
**SALL4 expression in bone marrow recovery. (a)**: H&E stain sections of bone marrows from Control (non-irradiated mice) or 3 days and 6 days post-sublethally irradiated mice. **(b)** SALL4 expression levels by RT-PCR correlate with bone marrow cellularity seen in the top (n = 3).

**Figure 3:**
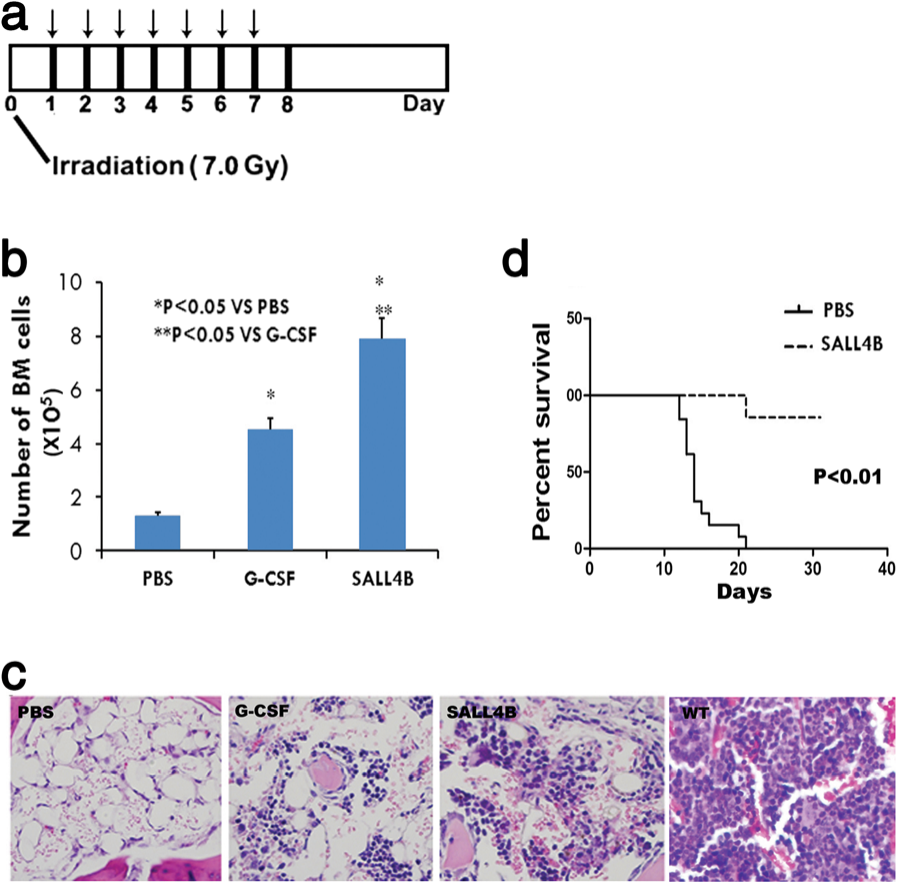
**TAT-SALL4B treatment increases the bone marrow regeneration and survival in lethally irradiated mice. (a)**: Strategy of irradiation and injection for bone marrow regeneration assay. **(b)**: Total bone marrow cell counts (n = 8). **(c)**: Histological staining of bone sections shows drastically enhanced cellularity of bone marrow in SALL4B and G-CSF group as compared to PBS or WT control. **(d)**: Survival curve of lethally irradiated mice after 7 days treatment of TAT-SALL4B (n = 7) or PBS (n = 10).

As previously shown by our group, SALL4 expression is seen in hematopoietic CD34 + [21], but not CD34- cells. Markedly elevated levels of SALL4 were detected at the early phase of bone marrow recovery after ablation and expression levels were decreased as bone marrow cellularity increased (Figure 2). This study indicates that upregulation of SALL4 may play a role of bone marrow recovery. We then tested the impact of TAT-SALL4B on the growth of residual bone marrow cells in vivo after ablation. A series of experiments were carried out in order to determine if the purified TAT-SALL4B fusion protein had the ability to regenerate bone marrow production *in vivo.* TAT-SALL4B protein, G-CSF, or PBS was intraperitoneally injected into mice for seven consecutive days 24 hours after lethal irradiation (Figure 3a). The dose of lethal irradiation (7 Gy, gamma-ray) administered to the mice was able to kill more than 99% of the mouse bone marrow cells within two to three days. An average of 2 × 10^7^ whole bone marrow nucleated cells was obtained from flushing out both tibias and femurs from one wide type mouse. In the PBS group, the number of whole bone marrow cells per animal was 1.32 ± 0.21 × 10^5^ at day 8 after irradiation. As consistent with previous reports, G-CSF increased the number of whole bone marrow cells by ~3-4 fold (4.51 ± 0.47 × 10^5^) [22]. The increase was over 6-fold (7.91 ± 0.83 × 10^5^) in the SALL4B group compared to the PBS control. These data suggest that SALL4B has a greater effect on boosting the proliferation of bone marrow cells after irradiation compared to G-CSF (Figure 3b). To further confirm our cell count data, we analyzed the histological sections from the various treatment groups 8 days after irradiation. In contrast to the PBS group, in which there were only very few cells, mainly marrow stromal cells left in mouse bone marrow cavity, the cellularity of the bone marrow was dramatically enhanced by SALL4B treatment, similar to that in the G-CSF treated animals (Figure 3c). Furthermore, we detected the existence of TAT-SALL4B in the bone marrow cells of mice by flow cytometry and immunofluorescent staining (Additional file 1: Figure S1). This demonstrated the cells that repopulating the marrow cavity were actively expressing the TAT-SALL4B protein. An additional control with TAT-GFP was used to exclude the possibility that the observed mouse bone marrow regeneration would have resulted from an effect of TAT. We expressed and purified TAT-GFP fusion protein using the same method utilized for TAT-SALL4B and compared the function of TAT-GFP to PBS in lethally irradiated mice. The results showed that there was no difference in the total number of bone marrow cells between the two groups (Additional file 1: Figure S2) suggesting TAT had no impact on the proliferation of bone marrow cells, and the SALL4B portion of the fusion protein accounted for the regeneration of mouse bone marrow.

### Radioprotection by TAT-SALL4B

To determine if the enhanced growth of residual marrow cells had a functional impact, we performed an animal survival assay after lethal irradiation. TAT-SALL4B treatment significantly increased the survival of mice 24 hours after 8 Gy lethal irradiation, a dose by which the mouse usually dies within 30 days. As depicted in Figure 3d, the cumulative actuarial 30-day survival rate in SALL4B is 85.7%, compared to 0% in the PBS control group. These data were consistent with the previous observation of improved bone marrow cellularity in TAT-SALL4B treated mice. An interesting note is that this radioprotection effect was achieved by post-irradiation administration of SALL4B, which is different from G-CSF treatments where radioprotection is effective only by administration before or within two hours after irradiation injury [23].

### TAT-SALL4B boosts HSC/HPC in injured mouse bone marrow

**Figure 4:**
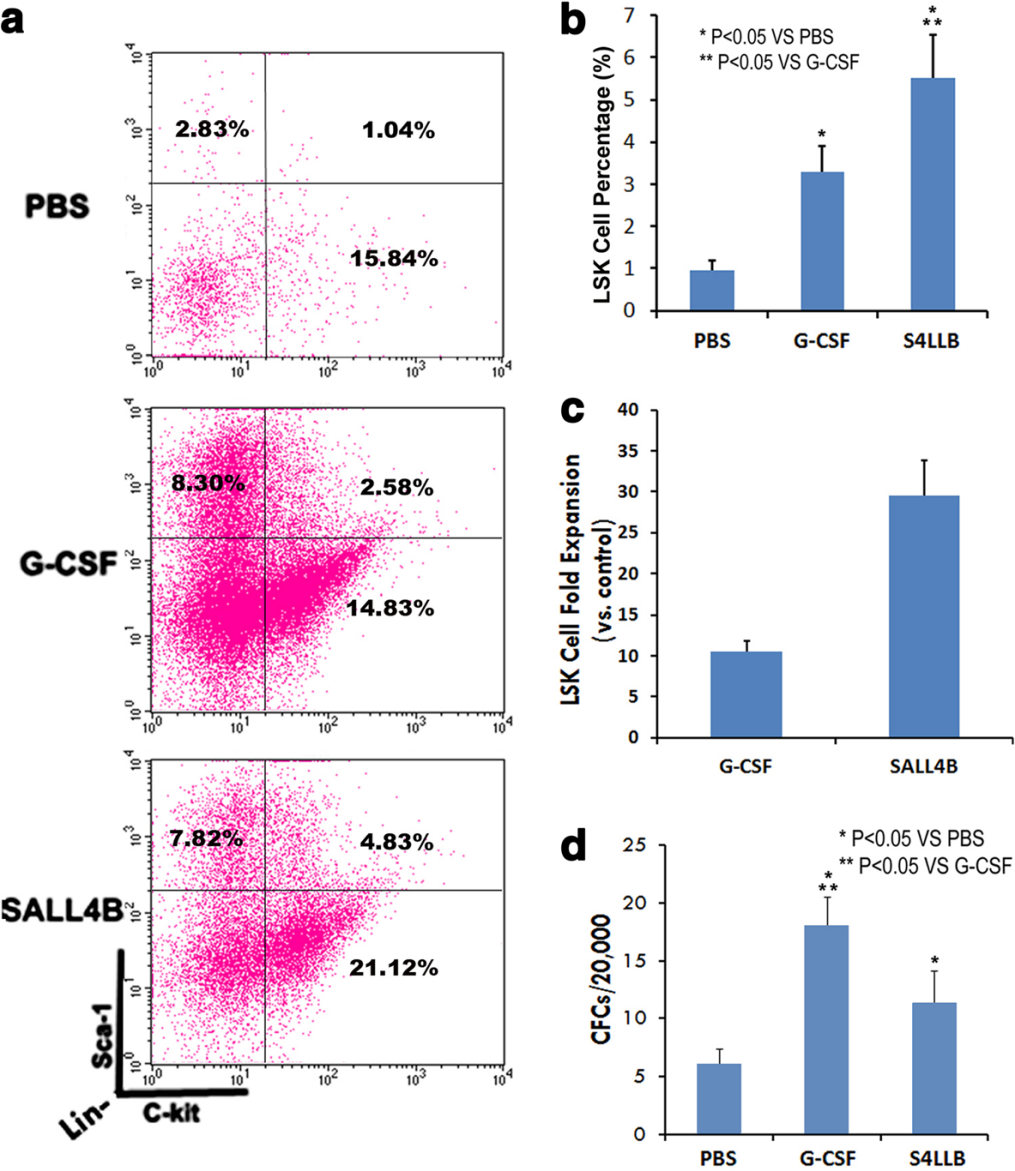
**TAT-SALL4B augments the content of hematopoietic precursor cells in bone marrow. (a)**: Representative pictures from flow cytometry analysis of LSK cell in bone marrow. *Note: The diagraphs presented were from Lin- cells, the listed LSK percentages is calculated to the whole bone marrow cell (both Lin + and Lin- cells included).***(b)**: The percentage of LSK cell in bone marrow TAT-S4LLB treated animals is significant higher than G-CSF and PBS control group (n = 5). **(c)**: Fold increase of bone marrow LSK cell in G-CSF and SALL4B group compared to control (n = 5). **(d)**: CFCs numbers using whole bone marrow cells of PBS, G-CSF or SALL4B treated mice (n = 3).

We then investigated the impact of TAT-SALL4B on the expansion of residual HSCs/HPCs in detail after ablation. Compared to the control group (0.94 ± 0.24%), LSK cell percentage was significantly higher in the G-CSF group (3.29 ± 0.62%). More importantly, the LSK cell percentage in the SALL4B group (5.52 ± 1.02%) was even higher than that in the G-CSF group (Figure 4a,b). The total fold increases (vs. control) of HSCs number (Whole BM number times LSK%) in mouse bone marrow were ~10 fold and ~30 fold in the G-CSF and SALL4B treated group, respectively (Figure 4c).

In order to analyze the number of progenitor cells in these bone marrow cell populations, CFC assays were conducted. In parallel with flow cytometry (data not shown), both G-CSF- and TAT-SALL4B-treated mice showed significantly higher bone marrow CFC content than mice treated with PBS and the G-CSF-treated mice had the highest bone marrow CFC content overall (Figure 4d). This is consistent with the observation of a biased effect by G-CSF toward lineage-restricted progenitors [22].

### TAT-SALL4B treatment increases short-term engraftment in HSCT

**Figure 5:**
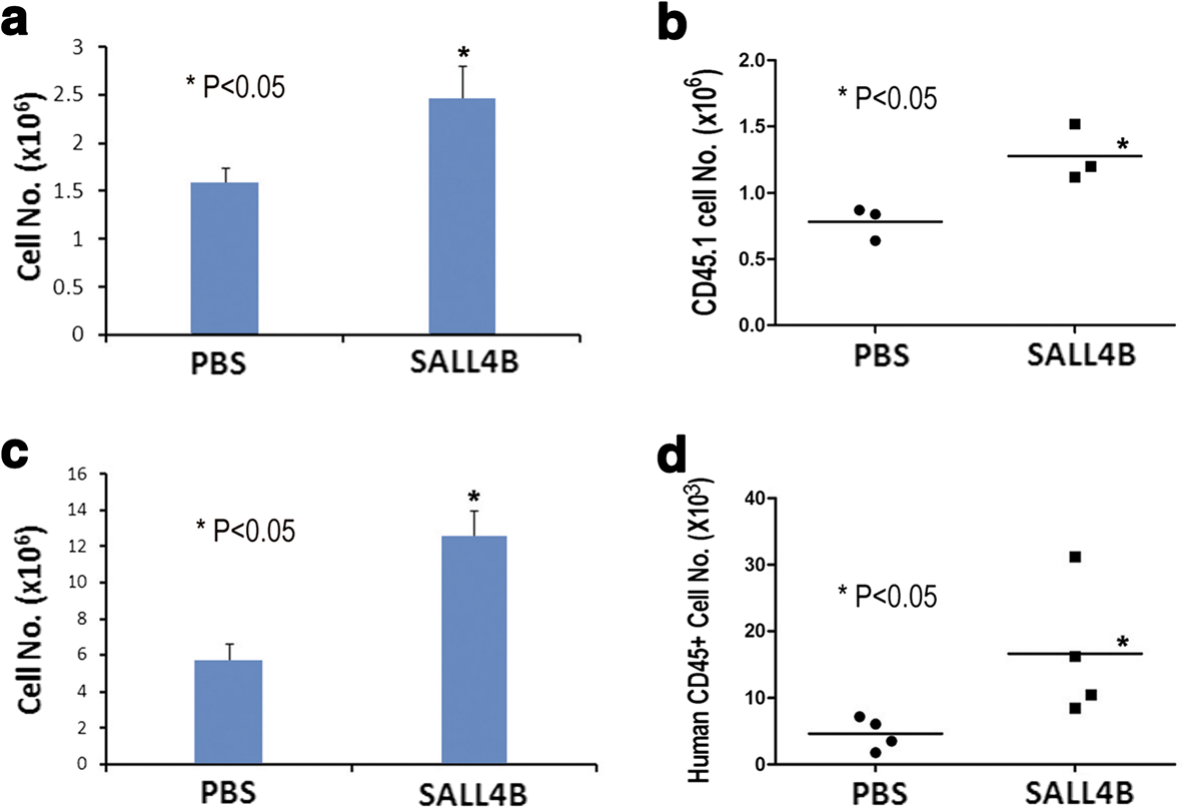
**TAT-SALL4B treatment increases the short-term engraftment of mouse or human cells in transplantation.** 400,000 c-kit positive bone marrow cells of CD45.1 B6/SJL mice were injected to lethally irradiated CD45.2 C57BL/6 mice, TAT-SALL4B injection augmented the total bone marrow **(a)** and donor CD45.1 cell numbers **(b)** in the recipient mice (n = 3). Similar results were observed in transplantation of human CB CD34+ cells into NOD/SCID mice. The total bone marrow cell number **(c)** and human CD45+ cells **(d)** in NOD/SCID mice were increased by TAT-SALL4B protein treatment (n = 4).

One major disadvantage of using human cord blood is that the absolute number of HSCs/HPCs in hUCB is much lower than that in bone marrow, which significantly delays engraftment [6]. We next tested if TAT-SALL4B was able to increase the proliferation of transplanted donor cells in the host. First, we used the mouse CD45.1/CD45.2 transplantation system. We enriched mouse BM precursor cells by c-Kit positive selection from CD45.1 mice and transplanted into lethally irradiated CD45.2 mice. At day 8, the mice in the SALL4B group had higher numbers of total bone marrow cells than those in the control group (2.44 ± 0.36 × 10^6^ vs. 1.58 ± 0.16 × 10^6^; P < 0.05, Figure 5a). When only donor cells (CD45.1+) were analyzed, the results showed that the SALL4B group had more donor cells compared to the controls (1.28 ± 0.21 × 10^6^ vs. 0.78 ± 0.13 × 10^6^; P < 0.05, Figure 5b), although the percentage of donor cells in the bone marrow was not different between the SALL4B group and control (data not shown).

In addition, we also tested the effect of the SALL4B protein on the short-term engraftment of human cord blood CD34+ cells when introduced into sublethally irradiated NOD/SCID mice. In parallel to the results from the CD45.1/CD45.2 transplantation system, the total number of bone marrow cells was significantly increased at 14 days after transplantation in the SALL4B treated animals compared to the PBS controls (12.5 ± 1.50 × 10^6^ vs. 5.67 ± 0.94 × 10^6^; P < 0.05, Figure 5c). Furthermore, the percentage of human cells as identified by human CD45 antibody in mouse bone marrow of SALL4B protein treated mice was greater than that of control, although this was not statistically significant (0.15 ± 0.11% vs. 0.085 ± 0.05% , P = 0.07, Additional file 1: Figure S3). However, when the total increase of whole bone marrow cells is taken into account, the increase of human CD45+ cells in the bone marrow is statistically significant in the SALL4B group when compared to controls (16.6 ± 10.2 × 10^3^ vs. 4.65 ± 2.45 × 10^3^; P < 0.05, Figure 5d).

These data prove that TAT-SALL4B treatment can promote proliferation of the host mouse bone marrow not only in a myeloablative condition (lethal irradiation) but also in a non-myeloablative condition (sublethal irradiation). Also, TAT-SALL4B can enhance the proliferation of donor cells after transplantation. Recent studies have shown that homing is an important mechanism for the increase of donor cell number in transplantation studies. In order to evaluate if the SALL4B protein has an effect on the homing activity of donor cells, we conducted further experiments using the CD45.1/CD45.2 transplantation system. In these trials, the recipient mice were intraperitoneally injected once with TAT-SALL4B after transplantation then sacrificed 24 hours later for analysis. Our results showed that there is no difference regarding the total number of bone marrow cells and donor cells between the SALL4B treated and control groups (Additional file 1: Figure S4). Therefore, we believe that the increased short-term engraftment in both the mouse-mouse and human-mouse transplantation studies was most likely related to the expansion of donor cells in the host and not their homing activity

### TAT-SALL4B injection enhances long-term repopulating capacity of human cord blood CD34+ cells in NOD/SCID mice

**Figure 6:**
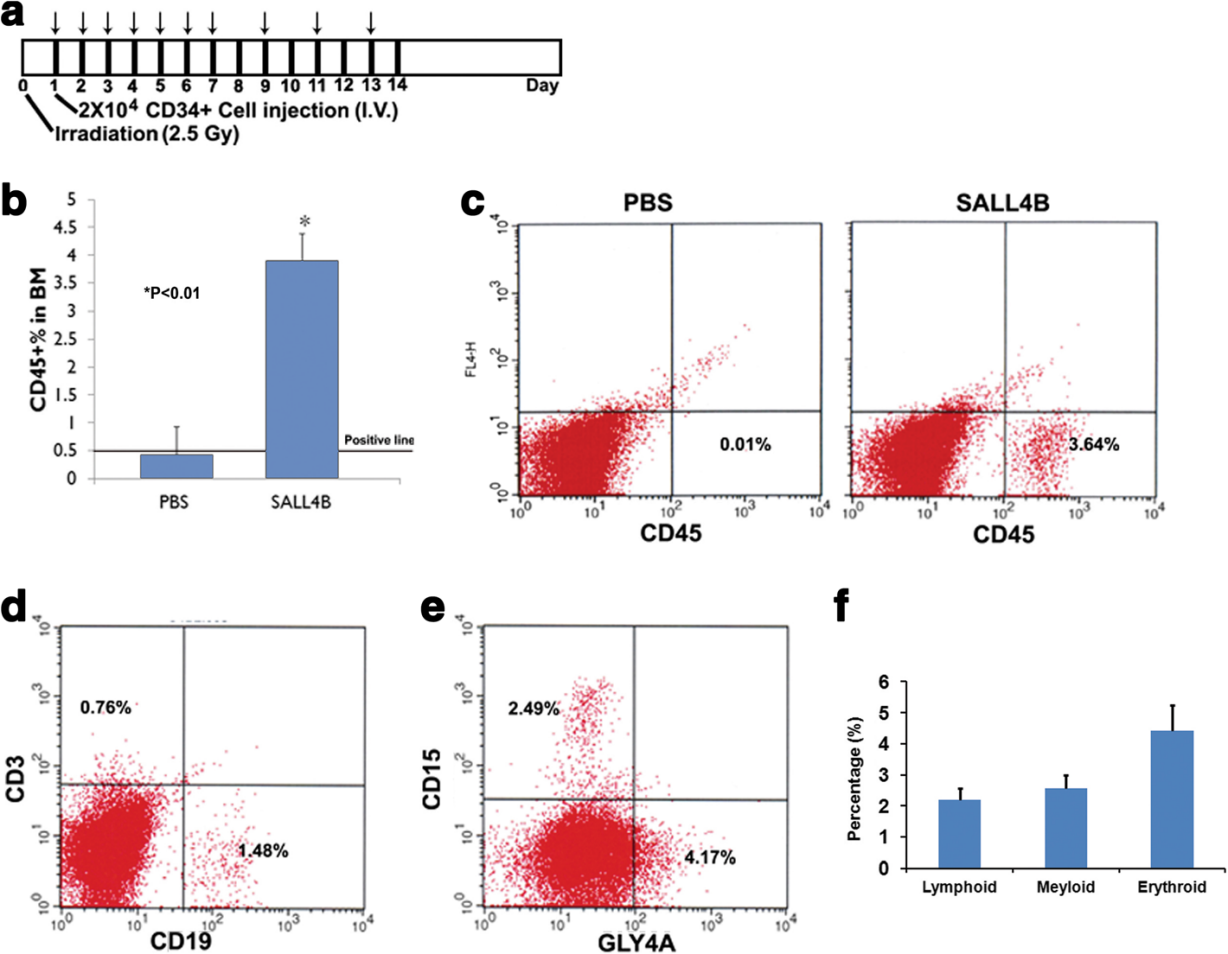
**TAT-SALL4B enhances the long-term engraftment of human cord blood CD34+ cells in NOD/SCID mice. (a)**: 20,000 human cord blood CD34+ cells were transplanted into NOD/SCID mice 24 hours after sublethal irradiation (2.5 Gy). Mice were treated with 2 μg TAT-SALL4B protein or PBS for 2 weeks (daily for the first week and once every other day for the second week). **(b)**: Percentages of human CD45+ cells in mouse bone marrow at sixteen weeks after transplantation in PBS and SALL4B treated group (n = 3). **(c)**: Representative flow cytometry results of human CD45+ cell percentage in mouse bone marrow. **(d)**: Representative multilineage differentiation of long-term engrafted human cells in NOD/SCID mouse. **(e)**: Percentages of lymphoid, myeloid and erythroid lineage from human donor cells.

Facilitation and maintenance of the long term engraftment of HSCs is of high importance in HSCT, which correlates with the success of hematological reconstitution and clinical outcome in patients after HSCT. We have observed that the TAT-SALL4B protein was able to increase the short-term engraftment of donor cells in both the mouse-to-mouse and human-to-mouse transplantation models. To address whether post-transplant administration of TAT-SALL4B is also capable of facilitating the long term engraftment of human HSCs, NOD/SCID mice were injected with human cord blood CD34+ cells via tail vein (Figure 6a). and received TAT-SALL4B or PBS intraperitoneally. Sixteen weeks after transplantation, there was a significant higher percentage of human CD45+ cells in the bone marrow of TAT-SALL4B treated mice compared to the control group (3.90 ± 0.36% vs. 0.43 ± 0.34%, P < 0.05, Figure 6b,c). Furthermore, these cells were able to differentiate *in vivo* into various blood lineages including lymphoid, myeloid, and erythroid lineages (Figure 6d,e). Of note is that SALL4B protein treatment may be able to reverse the higher lymphoid (versus myeloid) reconstitution usually observed in human cord blood HSCs transplantation in NOD/SCID mice [24]. This demonstrated that the TAT-SALL4B treatment does not alter the normal differentiation program of human HSCs in vivo and is consistent with our previous results using SALL4B over-expressed HSCs/HPCs by lentivirus transduction.

In order to determine if SALL4B protein could also affect tissues other than hematopoietic cells, we examined all of the major organs and found there were no any abnormalities by histology studies (data not shown). We also performed long-term follow up in a small number of mice receiving TAT-SALL4B post transplants. As shown in Additional file 1: Table SI, we monitored the animals throughout the study for body weight and hematopoietic parameters. No tumors occurred in different strains including C57Bl/6, B6/SJL and NOD/SCID. Some mice have been observed for more than 18 months since TAT-SALL4B administration with no tumor formation.

### N-terminal 12 amino acids are essential for SALL4B to stimulate the expansion of HSCs/HPCs

**Figure 7:**
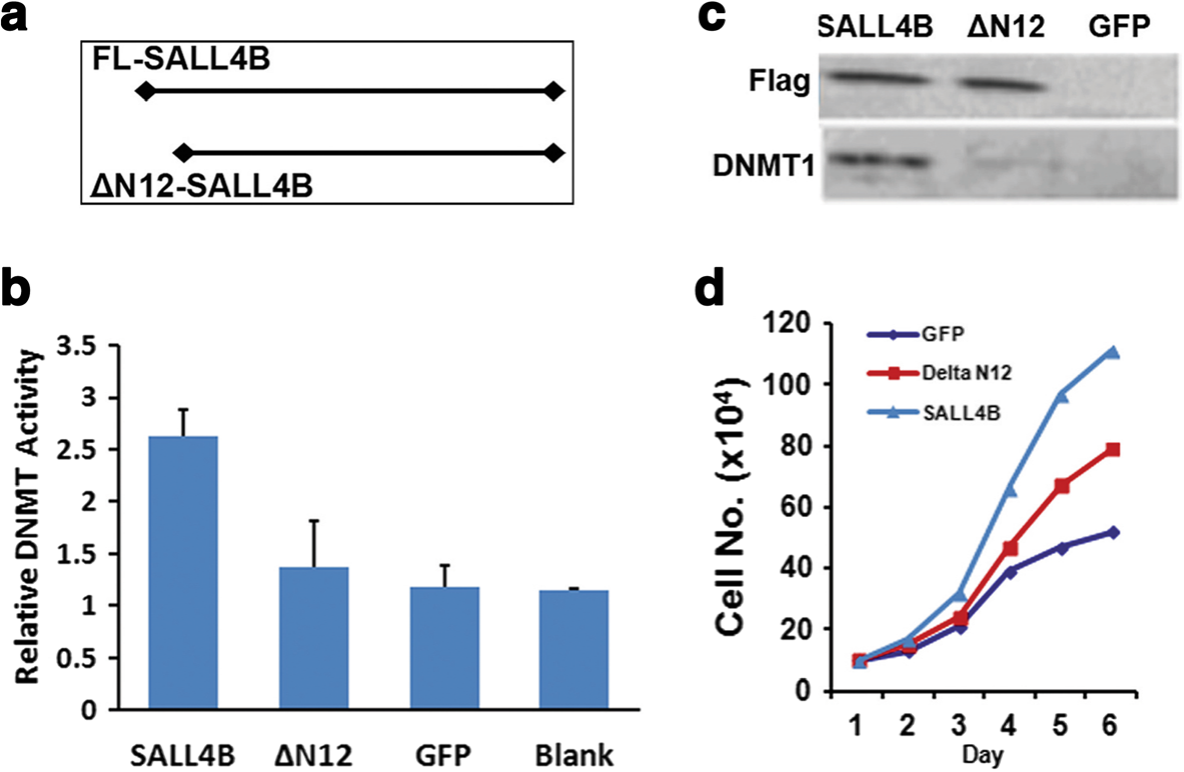
**Deletion of the N-12 aas markedly reduces SALL4B mediated LSK cell expansion. (a)** Full length (FL) and N-terminal 12 aas deleted (δΝ12) SALL4B protein. **(b)** N-12 aas are essential for recruiting DNMT activities. **(c)** IP in LSK cells shows N-12 aas are required for SALL4B/DNMT1 interaction. **(d)** Growth curve of mouse LSK cells transduced with FL-SALL4B, δΝ12-SALL4B or GFP lentivirus construct.

DNMT mediated epigenetic modifications are important for the self-renewal of hematopoietic stem cells. We have found that SALL4 actively recruits DNMT epigenetic modifiers (DNMT1, 3A, 3B, 3 L and MBD2) to target genes and leads to their inactivation. Downregulation of endogenous SALL4 expression led to accelerated cell differentiation of HSCs/HPCs [17]. Our previous studies also indicate that the SALL4 N-terminal sequence is essential for its interactions with DNMTs [25]. We conducted studies to further illustrate that the SALL4 N-terminal sequence is essential for its interactions with DNMTs. We generated an N-terminal 12 amino acid (aas) (N12) deleted SALL4B lentivirus construct (ΔN12-SALL4B) (Figure 7a). Deletion of the N-12 amino acids (aas) in SALL4B (ΔN12-SALL4B) strikingly diminished its physical interactions with DNMTs in mouse LSK cells resulting in a reduction of methyltransferase activity to a level that was comparable with that of a negative control (Figure 7b-c). When ΔN12-SALL4B was introduced to the HSC/HPC cells, it strikingly diminished the induction of HSC/HPC expansion unlike the SALL4B (Figure 7d).

## Discussion

Recently, we have demonstrated that bone marrow HSC/HPC transduced to express SALL4A or SALL4B are able to achieve high levels of *ex vivo* expansion without loss of their long-term engraftment capability. To evaluate the in vivo effect of SALL4 on the expansion of hematopoietic precusor cells in transplantion, we generated a CPP SALL4B fusion construct, TAT-SALL4B and expressed it in insect cells. Protein transduction utilizing CPP might overcome the limitations of lentiviral vectors, and has recently been in wide use [26-28]. There are multiple phase 2 clinical trials using the CPP approach as a systemic or topical delivery system and these include NCT00451256 for c-myc (prevention of undesirable cell proliferation in coronary artery bypass grafts) and PsorBan for cyclosporine andmNCT007885954 for a PKCδ inhibitor (the treatment of acute myocardial infarction).

We found that TAT-SALL4B was able to enhance the hUCB HSC/HPC engraftment in NOD/SCID. In our study, after treated with TAT-SALL4B protein, both short-term and long-term engraftment of human cells were significantly enhanced in hUCB CD34+ cell transplanted NOD/SCID mice. Notably, for short-term engraftment, although the percentage of human cells in mouse bone marrow did not show much difference between SALL4B and PBS treated animals, the absolute number of human cells was significantly increased after SALL4B treatment. Our studies indicate that the increased engraftment (absolute number of human cells) was likely a result of the direct expansion of donor cells in bone marrow by SALL4B treatment rather than enhanced homing of donor cells.

Interestingly, even though as low as 20,000 human CB CD34+ cells were used for the transplantation, at 4 months after transplantation, there were still a significant portion of human cells existing in the bone marrow of SALL4B treated mice as compared to almost no human cells for the control. Our results may have some therapeutic value in term of the low dose of stem cells in human UCB transplantation. Numerous clinical studies have consistently demonstrated that the total nucleated cell (TNC) and CD34+ cell doses in cord blood grafts are highly correlated with the rate of neutrophil and platelet engraftment, as well as the incidence of graft failure and early transplant-related complication [8]. Because of this, only ~10% of all cord blood specimens can be currently used. It is possible that the introduction of SALL4B to previously unusable cord blood units due to low TNC or CD34+ cell counts may be used in future therapies.

Drugs which promote rapid hematopoietic recovery would address the major cause of morbidity and mortality. G-CSF or derived drugs target specifically the hematopoietic system and prolong the median survival time of lethally irradiated mice [23]. However, no survival advantage is observed when mice receive G-CSF 24 hour’s post-TBI [23]. An increased time window after TBI would be advantageous in a nuclear emergency setting where healthcare provider times may be at a premium and they cannot administrate patients right away. In our study, we found that SALL4B injection dramatically regenerated the bone marrow in mice after irradiation, suggesting the potential radioprotective effect of SALL4B. In addition, this effect is achieved by injection 24 hour post-irradiation. Additionally, the TAT-SALL4 could potentially be used for increasing the therapeutic index of radiation therapy regimens for cancer patients by reducing the hematologic toxicity of ionizing radiation, enhancing bone marrow stem cell engraftment, and treatment of aplastic anemia.

Our studies also observed the stimulatory effect of SALL4B on bone marrow cells in both lethal and sub-lethal irradiation in mice. Since the bone marrow cell numbers are not obviously affected in wild type animals receiving SALL4B treatment (Additional file 1: Figure S2), this suggests that SALL4B protein may only boost the proliferation of bone marrow cells in pathological conditions when the bone marrow microenvironment allows more cells' occupation for the recovery. When the bone marrow recovers to a normal condition, the SALL4B will not cause a hyper-proliferation problem which is related to tumorigenesis. This is very important for safety issues. In fact, in order to address the concerns about safety, we monitored the animals throughout the study for body weight and hematopoietic parameters. No tumors occurred so far in different strains including C57Bl/6, B6/SJL and NOD/SCID, and the longest time since mice received SALL4B is over 18 months.

The mechanism underlying the stimulating effects on HSC/HPC expansion is still largely unknown. SALL4 is highly expressed and plays an important role in embryonic stem cells, primitive germ cells, HSCs/HPCs and acute leukemia [17,19,20,29]. Aside from the gatekeeper role in embryonic development and pluripotency of inner cell mass in blastocyst [19,20,30,31], SALL4 is essential for primordial germ cell survival [32]. Recent studies have suggested that hematopoiesis may be initiated from migrating germ cells [33,34] and SALL4 as a stem cell marker could be useful in further investigating this notion. Recently, we have found that SALL4 actively recruits DNMT epigenetic modifiers to target genes and lead to their inactivation. We further demonstrated that the N-terminal 12 amino acids were critical for SALL4B binding to DNMTs and the consequent expansion of HSCs in vitro. In the future, we will explore to shorten the protein based on these results and test their activity in in vivo expansion of bone marrow stem cells. In addition, bone marrow niche cells such as endothelial and perivascular cells [35] may also uptake TAT-SALL4B protein and contribute to the in vivo expansion of HSCs/HPCs in present study.

In conclusion, our data demonstrated that TAT-SALL4B protein from insect cells promoted hematopoietic recovery after lethal and sub-lethal irradiation. Furthermore, TAT-SALL4B treatment was able to enhance both the short-term and long-term engraftment of human UCB cells in NOD/SCID mice and the mechanism is likely related to the in vivo expansion of donor and recipient cells in the bone marrow. TAT-SALL4B protein could become an attractive candidate to enhance the engraftment of human UCB cells in hematopoietic stem cells transplantation and facilitate the hematopoietic recovery after radiation injury.

## Material and methods

### Purification of TAT-SALL4B

Sf9 insect cells (ATCC, Manassas, VA, USA) were transfected with a baculovirus expression construct containing either the human SALL4B sequence, or a GFP control, each with a c-terminal 6x His fusion tag. To recover recombinant protein, cells were lysed in lysis buffer [50 mM Na2HPO4/NaH2PO4 (pH 7.4), 300 mM NaCl, 20 mM NEM, 0.2% Triton X-100] containing 20 mM imidazole. His-tagged proteins were eluted in the lysis buffer containing 300 mM imidazole and blotted for detection with SALL4 or GFP antibody. Fractions containing the desired protein were pooled and dialyzed in IMDM overnight.

### Promoter assay

Promoter luciferase assays were performed with the Dual-Luciferase Reporter Assay System (Promega, Madison,WI, USA) as described previously [20]. Briefly, MCF-7 cells were transfected with the SALL4B or OCT4 promoter reporter plasmid [20] using Lipofectamine 2000 (Invitrogen, Grand Island, NY, USA). Five hours later, cells were changed to fresh medium and TAT-SALL4B proteins or BSA were added. The next day, cells were analyzed after treated with TAT-SALL4B Protein or BSA for 3 times. The data are represented as the ratio of firefly to Renilla luciferase activity (Fluc/Rluc). These experiments were performed in duplicate.

### Animals

Animals used includes three different strains C57BL/6 (CD45.2), B6/SJL (CD45.1) and NOD/SCID mouse (The Jackson Laboratory, Bar Harbor, ME, USA). All animal procedures were approved by Stony Brook University Institutional Animal Care and Use Committee.

### Cell culture

Bone marrow cells were collected from C57BL/6 mice femur and tibia and magnetically sorted with Lineage, c-Kit and Sca-1 labeling kits from MACS (Miltenyi Biotec, Auburn, CA, USA) and cultured in DMEM supplemented with 10% FBS, 100 ng/ml mSCF, 6 ng/ml mIL-3 and 10 ng/ml hIL-6 (ProSpec, Rehovot, Israel). LSK (lineage-/c-Kit+/Sca-1) cells were plated in 24-well at 100,000 cells/well and treated with 20 nM TAT-SALL4B or BSA daily. Cells were counted every day for 6 days. In another experiment. the LSK cells were infected with full-length SALL4B, N-terminal 12 amino acids deleted SALL4B (ΔN12-SALL4B) or GFP lentiviruses overnight for 12–15 hours then recovered in culture medium.

### Bone marrow recovery analysis and survival test

Eight week old C57BL/6 mice received 7 Gy total body irradiation (TBI) at dose of 0.6 Gy/min. Twenty four hours later, 6 μg TAT-SALL4B protein, 2 μg G-CSF (as previously described [22]), 6 μg TAT-GFP protein or PBS was injected intraperitoneally daily for seven days. At day 8, bone marrow cells were counted and analyzed by flow cytometry. In addition, bone marrow tissue sections were prepared for Wright-Giemsa staining. For survival test, mice were given a single dose of 8 Gy TBI and received TAT-SALL4B or PBS in same pattern as mentioned above. Mice were monitored daily after irradiation for 30 days.

### Cell transplantation

There are two transplantation models utilized in our study: CD45.1/CD45.2 (mouse to mouse) and CD34+/NOD/SCID (human to mouse) models. Animals received 7 Gy TBI for C57BL/6 mice or 2.5 Gy TBI for NOD/SCID mice. 24 hours after irradiation, animal were injected with mouse 400,000 c-Kit + or 40,000 human CB CD34+ cells. For short-term engraftment analysis, transplanted mice were given 7 consecutive TAT-SALL4B protein or PBS injections for seven days. At day 8 or day 14, total bone marrow cells were counted and the donor cells in recipient mice were analyzed using mouse CD45.1 or human CD45 antibody. For long-term engraftment experiment, 20,000 human CB CD34+ cells were transplanted into NOD/SCD mice and 4 additional injections of proteins were conducted in the following week. Human cell content in the bone marrow was checked 16 weeks after transplantation.

### Homing assay

CD45.2 mice were irradiated (7 Gy) and transplanted with 400,000 CD45.1 mouse bone marrow c-Kit + cells 24 hours later. TAT-SALL4B protein or PBS was injected into CD45.2 mice right after transplantation. Animals were sacrificed for bone marrow cell collection 24 hours after transplantation. The percentage of CD45.1 cells in bone marrow cells were analyzed by flow cytometry and the absolute number of CD45.1 cells was calculated.

### CFC assay

20,000 bone marrow cells from TAT-SALL4B, G-CSF or PBS treated animals were suspended in MethoCult® (Stemcell Technologies) medium for CFC assay according manufacturer's instruction. A colony with more than 100 cells was counted as a positive colony.

### Flow cytometry

Phycoerythrin (PE), Fluorescein isothiocyanate (FITC), allophycocyanin (APC), or PerCP-Cy5.5-conjugated antibodies to human CD34, CD19, CD38,CD15, CD33, CD45, CD3 or mouse CD45.1, CD45.2, Lineage, Sca-1, c-Kit (BD Biosciences, San Jose, CA, USA) were used for flow cytometry analysis. Isotype control antibodies were used for compensating and gating when setting up the flow cytometer for each analysis.

### Quantitative RT-PCR

Quantitative RT-PCR (qRT-PCR) analyses were performed as reported previeously [20]. The primer sets for SALL4 were Forward: GCAGCCTCAGCAGCTACC, Reverse: GGGAGTTCACTGGAGCAC; and GAPDH were: Forward: ATACGGCTACAGCAACAGGG, Reverse: GCCTCTCTTGCTCAGTGTCC.

### Immunoprecipitation

HEK 293 cells were infected with Flag tagged SALL4B or ΔN12SALL4B lentivirus. Proteins were prepared with CelLytic™ MT Cell Lysis Reagent (Sigma-Aldrich, St Louis, MO, USA). Immunoprecipitations were performed by using Dynabeads® Protein G Immunoprecipitation Kit (Invitrogen) according to manufacturer’s instructions. Western blots were conducted with antibodies against Flag (Bethyl Laboratories, Montgomery, TX, USA) and DNMT1 (Novus Biologicals, Littleton, CO, USA).

### DNA methyltransferase activity assay

These experiments were carried out using the EpiQuik DNMT Activity/Inhibition Assay Ultra Kit (Epigentek, Farmingdale, NY, USA) following the manufacturer’s procedures. Firstly, nuclear proteins were extracted from tested cells using the EpiQuik™ Nuclear Extraction Kit (Epigentek). For immunoprecipitations preceding the assays, we used antibodies against HA or IgG control from Bethyl Laboratories.

### Statistical analysis

Results are reported as means ± SD. Values with *P* <0.05 were considered to be statistically significant.

## Competing interest

Yupo Ma is a scientific consultant to MarrowSource Therapeutics International LLC. The authors have no other relevant affiliations or financial involvement with any organization or entity with a financial interest in or financial conflict with the subject matter or materials discussed in the manuscript apart from those disclosed.

## Authors’ contribution

WL, JA and YP designed research; WL, JA and JY performed research and analyzed data; WL, JA and YP wrote the paper; and all authors critically reviewed and edited the paper. WL, JA, YY, JY, GZ, YJ, CA, LS, RL, XWD and YM revised and approved the final manuscript.

## Supplementary Material

Additional file 1: Table S1 Follow-up irradiated mice receiving TAT-SALL4B protein post-transplant. Click here for file
