# Supplementary material for: Enhancing bone marrow regeneration by SALL4 protein
Source: J Hematol Oncol. 2013 Nov 5;6:84. doi: 10.1186/1756-8722-6-84 (PMC3882884; doi:10.1186/1756-8722-6-84)
Supplement: Additional file 1: Table S1 — Follow-up irradiated mice receiving TAT-SALL4B protein post-transplant. [file 1756-8722-6-84-S1.docx]

**Supplemental Table and Figures**

**Table S1**

**Table S1. Follow-up irradiated mice receiving TAT-SALL4B protein post-transplant**

**
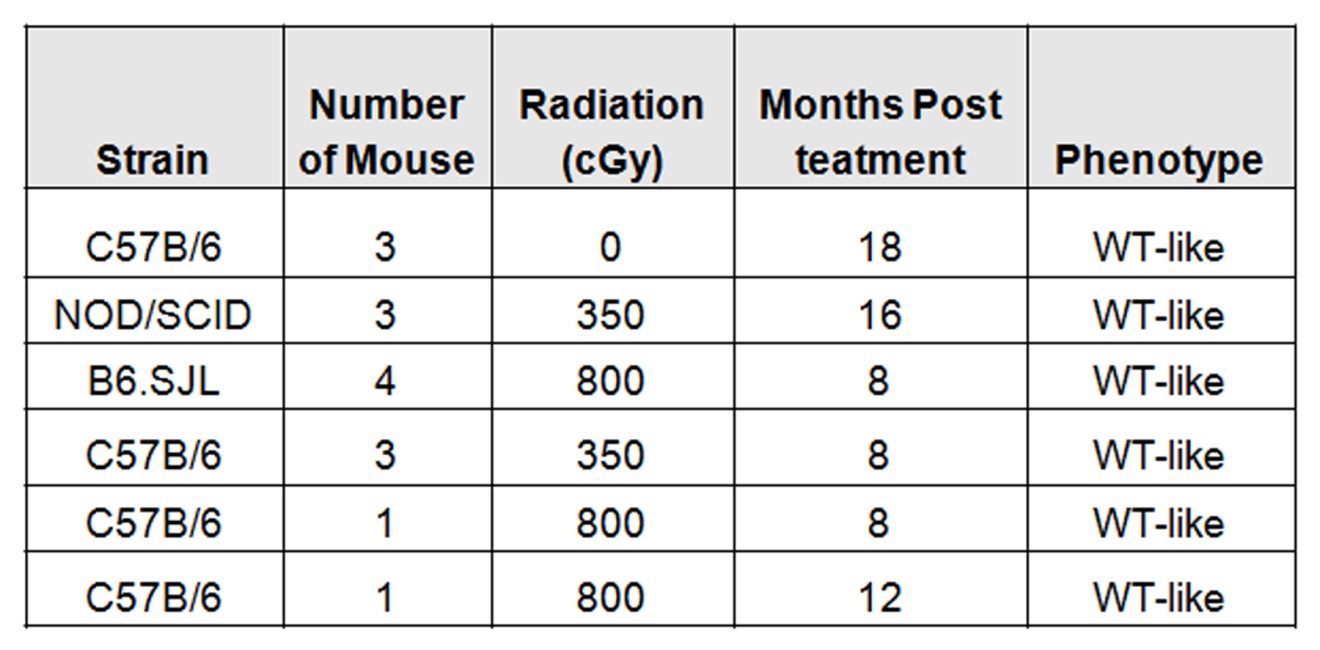
**

**Figure S1.
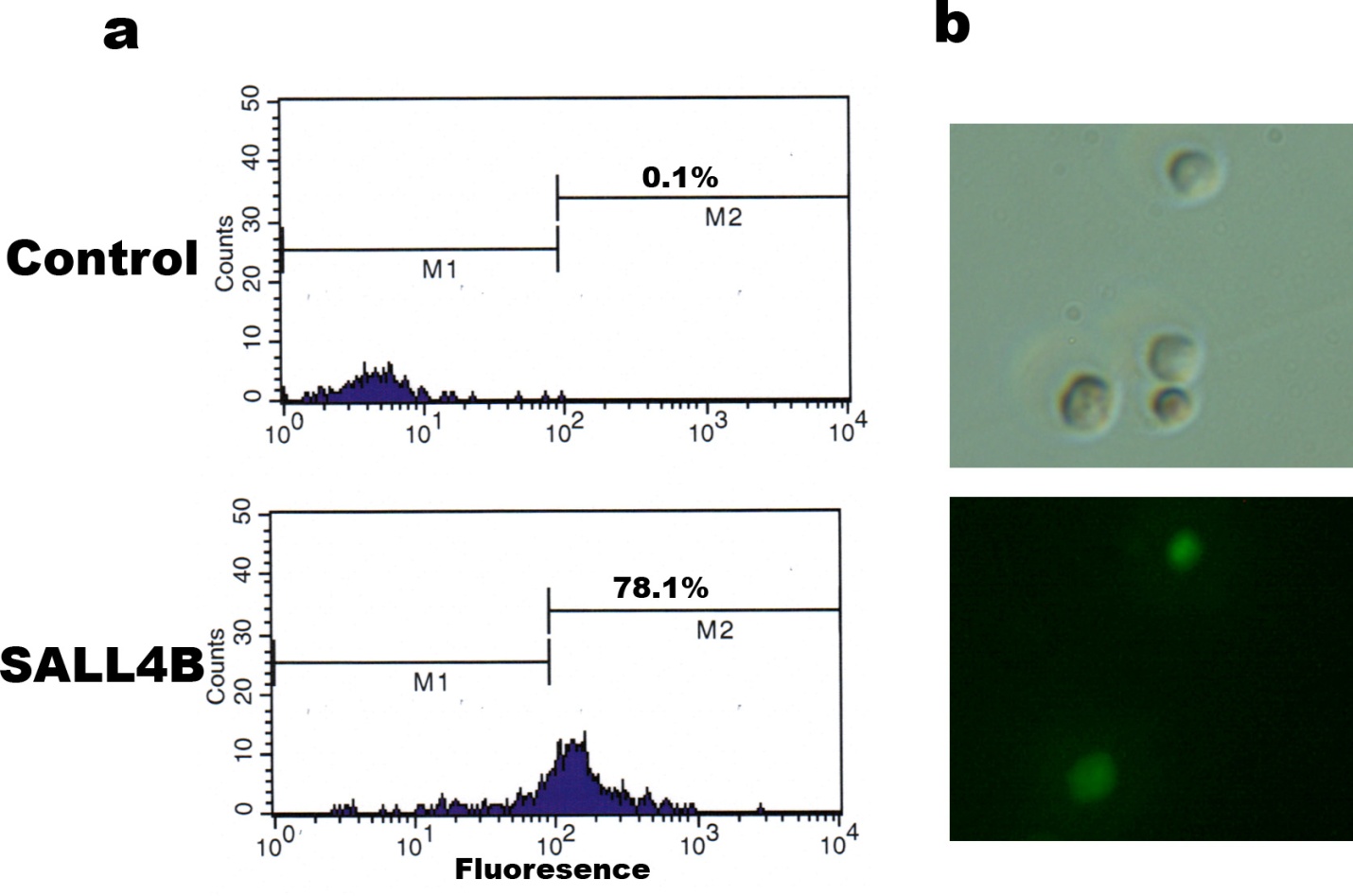
**

**Figure S1. Uptake of TAT-SALL4B protein by bone marrow cells.** Existence of TAT-SALL4B in bone marrow cells of mice receiving intraperitoneal injection of TAT-SALL4B as shown by flow cytometry analysis (a) and immunofluorescent staining (b) by anti-6xHis antibody.

**Figure S2.**

**Figure S2. TAT has no effect on bone marrow regeneration.** The number of total bone marrow cells in TAT-GFP treated mice is not different from PBS control (n=3).

**Figure S3.**

**
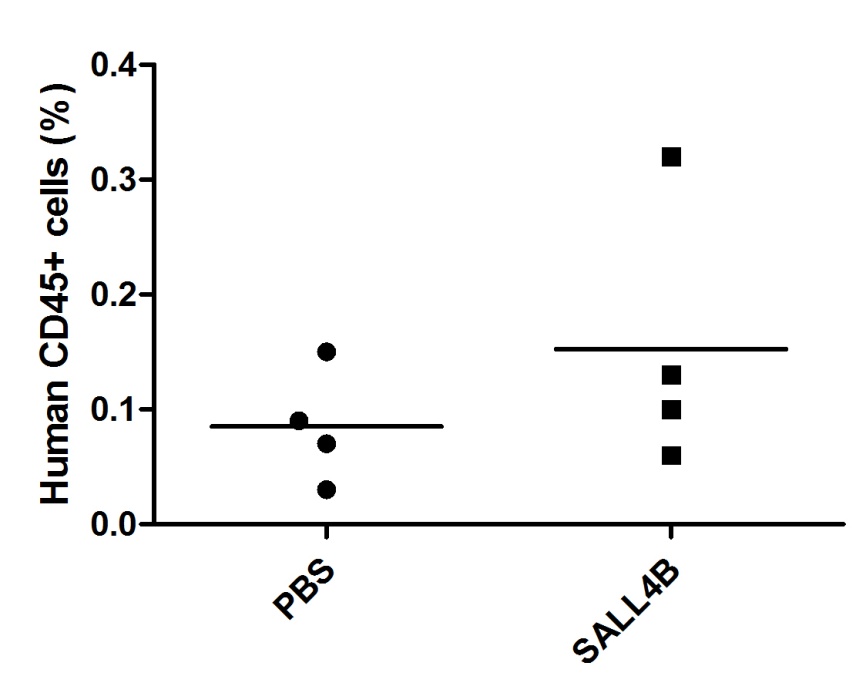
**

**Figure S3. TAT-SALL4B does not significantly increase the human cells ratio in short-term engraftment.** Sub-lethally irradiated NOD/SCID received 40,000 human CB CD34+ cell and were injected with TAT-SALL4B protein or PBS for 7 days. The percentage of human CD45+ cells in mice bone marrow in SALL4B group is higher but not statistically significant than PBS control at 14 days after transplantation.

**Figure S4**

**Figure S4. TAT-SALL4B has no effect on donor cell homing in transplantation.** The absolute numbers of donor CD45.1 cells in recipient CD45.2 mouse bone marrow are not different between SALL4B and PBS group 24 hours after transplantation (n=3).
